# Safety and efficacy of ciprofol for general anesthesia induction in elderly patients undergoing lumbar surgery

**DOI:** 10.3389/fmed.2025.1525973

**Published:** 2025-06-25

**Authors:** Gang Yao, Xiaochun Wei, Yu Zhong, Guofeng Liu, Yanzhuo Zhang, Yanhua Chen

**Affiliations:** ^1^Department of Anesthesiology, The First Affiliated Hospital of Guangxi Medical University, Nanning, China; ^2^Department of Anesthesiology, Liuzhou Workers’ Hospital, Liuzhou, China; ^3^Liuzhou Perioperative Neuroprotection Engineering Technology Research Center, Liuzhou, China; ^4^Liuzhou Key Laboratory of Anesthesia and Perioperative Neuroprotection, Liuzhou, China

**Keywords:** ciprofol, general anesthesia, anesthesia induction, geriatric lumbar surgery, elderly

## Abstract

**Objective:**

This study aimed to evaluate the safety and efficacy of ciprofol for anesthesia induction in elderly patients undergoing lumbar surgery.

**Methods:**

Sixty patients aged 65–80 years scheduled for elective lumbar surgery under general anesthesia were randomly divided into two groups (*n* = 30 each): ciprofol group and propofol group. The ciprofol group received intravenous ciprofol 0.4 mg/kg combined with sufentanil 0.4 μg/kg, while the propofol group received propofol 2 mg/kg combined with sufentanil 0.4 μg/kg for anesthesia induction. Hemodynamic parameters including bispectral index (BIS), systolic blood pressure (SBP), diastolic blood pressure (DBP), mean arterial pressure (MAP), cardiac index (CI), pulse pressure variation (PPV), systemic vascular resistance index (SVRI), and stroke volume index were recorded during induction. Time to achieve target anesthetic depth was also evaluated. Adverse events including movement, lacrimation, hypotension, hypertension, bradycardia, and coughing during induction were documented.

**Results:**

Both groups showed comparable time to achieve target anesthetic depth and similar trends in hemodynamic changes. However, the ciprofol group demonstrated significantly lower incidence of hypotension compared to the propofol group (20% vs. 63%, *P* < 0.05), with reduced norepinephrine consumption. The incidence of other adverse events showed no significant differences between groups.

**Conclusion:**

Ciprofol demonstrates comparable safety and efficacy to propofol for anesthesia induction in elderly patients undergoing lumbar surgery, with superior hemodynamic stability, supporting its clinical application in geriatric lumbar surgery.

**Clinical trial registration:**

The trial was registered, before patient enrollment, in the Chinese Clinical Trial Registry (www.chictr.org.cn) (Clinical trial number: ChiCTR2300069858, https://www.chictr.org.cn/showproj.html?proj=192839, principal investigator’s name: Gang Yao, date of registration: 28/03/2023).

## 1 Introduction

Anesthesia induction constitutes a critical phase in perioperative management, particularly among elderly surgical populations where hemodynamic instability may precipitate severe complications. Propofol, currently the most widely used intravenous anesthetic for induction, offers rapid onset and high-quality recovery but exhibits significant cardiovascular depression, particularly pronounced in elderly patients (1). Research indicates that elderly patients have a markedly higher risk of hypotension during propofol-induced anesthesia compared to younger patients (2, 3), attributed to age-related declines in cardiovascular compensatory mechanisms and increased susceptibility to fluid shifts (4–6). Ciprofol, a novel intravenous anesthetic first introduced in China in 2017, exhibits pharmacodynamic properties similar to propofol but with a reduced number of adverse events (7). While previous studies have demonstrated favorable efficacy and safety of ciprofol for anesthesia induction in healthy adults (8–10). However, evidence remains scarce regarding ciprofol’s application in elderly patients undergoing prolonged lumbar surgeries. Elderly patients undergoing lumbar surgery often present with varying degrees of cardiovascular comorbidities, The prone positioning required for lumbar procedures may further exacerbate hemodynamic fluctuations (11), making the choice of induction agent particularly crucial. Furthermore, age-related pharmacokinetic alterations, including decreased hepatic metabolism and altered volume of distribution (12), necessitate careful dose optimization. Current literature lacks robust data on ciprofol’s hemodynamic impact and optimal dosing strategies in this vulnerable population. This randomized controlled trial aims to compare the efficacy and safety of ciprofol versus propofol for general anesthesia induction in elderly patients (65–80 years) undergoing lumbar surgery, with primary emphasis on hemodynamic stability. The findings may establish evidence-based protocols for anesthetic management in geriatric orthopedic populations.

## 2 Materials and methods

### 2.1 General information

All patients in this study signed informed consent forms, and the protocol was approved by the hospital’s Ethics Committee (KY2023404). Participants were recruited between 1 April 2023, and 31 July 2023. Eligibility screening targeted patients aged 65–80 years scheduled for elective lumbar spine surgery under general anesthesia. Inclusion criteria comprised patients undergoing lumbar fusion with internal fixation, regardless of gender, with an American Society of Anesthesiologists (ASA) classification of grade II-III. Exclusion criteria included: Preoperative psychiatric or cognitive disorders; Long-term use of sedative medications (defined as regular intake of benzodiazepines, non-benzodiazepine hypnotics, barbiturates, or antipsychotics with sedative properties for ≥3 months prior to surgery); Obesity (body mass index [BMI] ≥ 28 kg/m^2^); Contraindications to anesthesia-related medications used in this study; Severe hepatic impairment (Child-Pugh score ≥ Class B) or renal impairment (serum creatinine > 2.0 mg/dL) prior to surgery. Refusal to participate. Additional exclusions applied to patients who developed severe allergic reactions during the study or withdrawal consent (either directly or via their legal representatives).

A randomized block design was employed for participant allocation, with a block size of four. Within each block, two participants were assigned to the experimental group and two to the control group. All participants were blinded to their group assignments. The anesthesia provider responsible for preparing the drugs was unblinded to group allocations but did not participate in any other study procedures or assessments. Endpoints evaluation and data collection were conducted by researchers who were blinded to the assignments.

### 2.2 Research methods

Upon entering the operating room, patients were positioned supine and monitored with standard anesthesia monitors, including three-lead electrocardiogram (ECG), heart rate (HR), pulse oximetry (SpO2 ), systolic blood pressure (SBP), diastolic blood pressure (DBP), mean arterial pressure (MAP), and bispectral index (BIS). Continuous hemodynamic parameters, including cardiac index (CI), pulse pressure variation (PPV), systemic vascular resistance index (SVRI), and stroke index (SI), were also monitored using the continuous non-invasive hemodynamic monitoring system (CNAP). Peripheral intravenous access was established, and 500 ml of hydroxyethyl starch electrolyte solution was slowly infused. Radial artery catheterization was performed under local lidocaine infiltration anesthesia, and after successful cannulation, invasive arterial pressure was continuously monitored via a pressure transducer. After preparation of anesthesia equipment, anesthetics, and resuscitation medications, mask oxygenation was initiated, followed by induction of anesthesia and endotracheal intubation using a video laryngoscope.

Anesthesia induction protocol: Anesthesia was induced with either ciprofol 0.4 mg/kg (Ciprofol Group) or propofol 2.0 mg/kg (Propofol Group), co-administered with sufentanil 0.4 μg/kg via controlled intravenous infusion over ≥30 s. Hemodynamic parameters and BIS were continuously monitored to titrate infusion rates, targeting BIS ≤60. The eyelash reflex was assessed at 5-s intervals post-infusion. If the eyelash reflex persisted or BIS was >60 at 30 s post-induction, supplemental doses (ciprofol 0.1 mg/kg; propofol 0.5 mg/kg) were administered. Induction failure was defined as BIS > 60 after three rescue doses. The modified observer’s assessment of alertness/sedation (MOAA/S) score was then recorded. Following loss of eyelash reflex, an MOAA/S score < 1, and BIS < 60, rocuronium 0.6 mg/kg was administered. Endotracheal intubation was performed after confirming adequate muscle relaxation. Dose reductions were applied for patients with comorbidities (e.g., cardiac dysfunction, pulmonary disease, or malnutrition).

Anesthesia maintenance: During surgery, propofol/ciprofol and remifentanil infusion rates were adjusted based on HR and BIS values. Sufentanil was intermittently administered as needed to maintain stable blood pressure and HR, with BIS values between 40 and 60.

During anesthesia induction, hypertension was defined as SBP > 20% above baseline (13), prompting an additional dose of the respective study drug. Hypotension was defined as SBP decreases > 20% below baseline (14) or MAP < 60 mmHg (15), treated with intravenous norepinephrine 40 μg. Tachycardia (HR > 110 bpm) required an additional dose of the study drug, while bradycardia (HR < 60 bpm) was managed with intravenous atropine 0.25 mg. In cases of allergic reaction, medication administration was stopped, and intravenous dexamethasone and calcium gluconate were administered. Unblinding conducted as necessary. In the event of anaphylactic shock or cardiac arrest, immediate unblinding was performed to guide resuscitation and appropriate treatment measures were implemented.

### 2.3 Observation indicators

Observation indicators were recorded at T0 (after the patient entered the operating room and received at least 5 min of calm oxygen inhalation) T1 (first loss of eyelash reflex with MOAA/S score < 1 and BIS < 60), and T2 (completion of endotracheal intubation). Monitored parameters included BIS, SBP, DBP, MAP, CI, PPV, SVRI, and SI. The time from drug administration to achieving target anesthesia depth (T1-T0) was recorded. Maximum and minimum values of BIS, SBP, DBP, MAP, and HR during induction were noted. Use of norepinephrine and atropine, as well as adverse events (body movement, tearing, hypotension, hypertension, bradycardia, and coughing), were recorded.

### 2.4 Statistical analysis

Statistical analysis was performed using SPSS 26.0. Continuous variables with normal distribution were presented as mean ± standard deviation (Mean ± SD), and compared using independent sample *t*-test. Non-normally distributed continuous variables were expressed as median (interquartile range) [M(P_25_–P_75_)] and analyzed using the Mann-Whitney U test. Categorical variables were described as frequency counts or percentages [n (%)] and analyzed using the chi-square (χ^2^) test or the Fisher exact probability method. Continuous variables with repeated measures are first tested for normality and sphericity, and repeated measures analysis of variance is used when conditions are met, otherwise generalized estimating equation (GEE) analysis is used. All statistical tests were two-sided tests, and the significance level was set to α = 0.05.

## 3 Results

**FIGURE 1:**
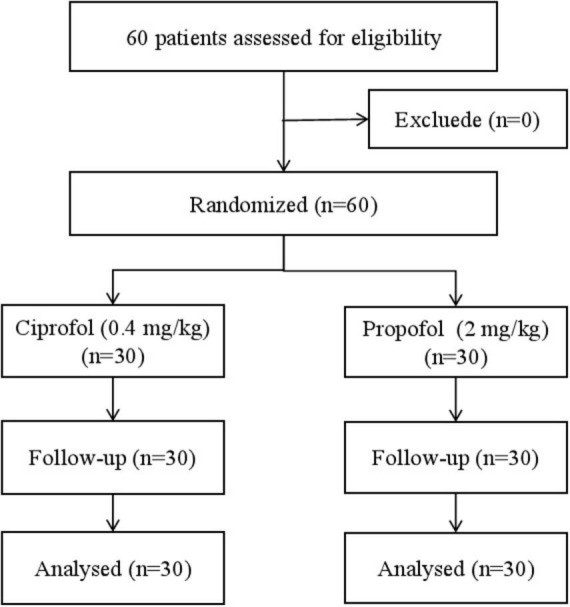
Flow diagram for the study.

A total of 60 patients successful underwent anesthesia induction and completed surgeries without complications (Figure 1). Baseline characteristics (age, BMI, gender, ASA classification, preoperative comorbidities) and hemodynamic parameters did not differ significantly between groups (Table 1).

**TABLE 1:** General information on research subjects.

Variable	Ciprofol (*n* = 30)	Propofol (*n* = 30)	*p*
Gender (M/F)	8 (26.7%)/22 (73.3%)	12 (40%)/18 (60%)	0.273
Age (year), *n*	70.5 (67.0–73.0)	69.5 (66.0–74.0)	0.923
ASA (II/III)	16 (53.3%)/14 (46.7%)	18 (60%)/12 (40%)	0.602
BMI (kg/m^2^)	23.4 (21.2–28.4)	23.4 (22.1–25.7)	0.894
Complication, *n*	14 (46.7%)/16 (53.3%)	16 (53.3%)/14 (46.7%)	0.606
Baseline BIS	95.00 (93.00–97.00)	94.50 (91.00–97.00)	0.760
Baseline SBP (mmHg)	164.50 (149.00–178.00)	164.50 (159.00–174.00)	0.539
Baseline DBP (mmHg)	78.00 (71.00–90.00)	79.50 (73.00–95.00)	0.391
Baseline MAP (mmHg)	111.00 (102.00–119.00)	111.50 (100.00–127.00)	0.515
Baseline CI (L/min/m^2^)	3.10 (2.70–3.50)	3.30 (2.90–3.70)	0.472
Baseline PPV (%)	5.00 (3.00–8.00)	5.50 (4.00–7.00)	0.870
Baseline SVRI (dyn⋅ s ⋅cm^5^⋅m^2^)	2923.03 ± 86.30	2886.80 ± 784.73	0.867
Baseline SI (mL/m^2^/min)	43.80 ± 10.74	43.69 ± 9.42	0.967

**FIGURE 2:**
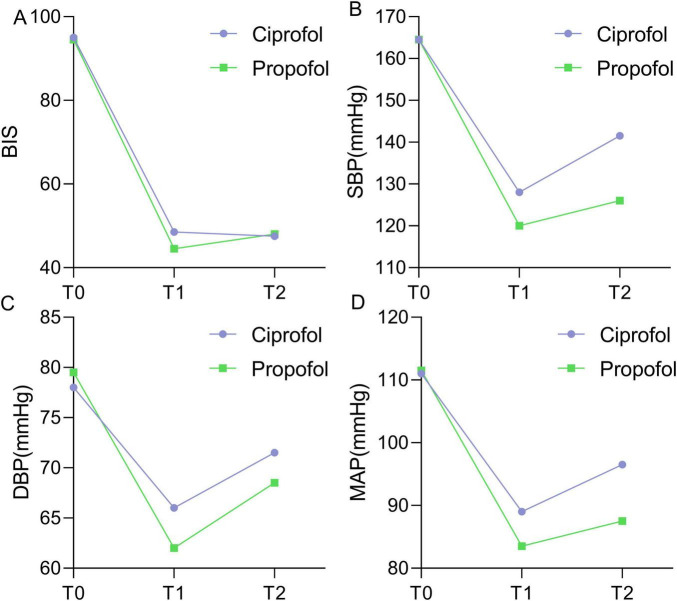
Changes of BIS **(A)**, SBP **(B)**, DBP **(C)**, and MAP **(D)** from T0 to T2, with purple for ciprofoll and green for propofol groups. T0: Before anesthesia induction after the patient entered the operating room and inhaled oxygen for at least 5 min; T1: the first eyelash reflex disappeared after the induction of anesthesia, when the MOAA/S score was <1 and the BIS was < 60; T2: when the endotracheal intubation was completed.

Bispectral index, SBP, DBP, and MAP showed downward trend from T0 to T1 and T2 in both groups, with SBP, DBP, and MAP reaching their lowest values at T1 (Figure 2). GEE analysis revealed significant time effects for BIS, SBP, DBP, and MAP (Table 2) and significant group-time interaction effect for SBP and MAP.

**TABLE 2:** Interaction effects of group and time on BIS, SBP, DBP, and MAP in the generalized estimating equation (GEE).

Parameter	BIS		SBP		DBP		MAP	
	*Waldχ^2^*	*p*	*Waldχ^2^*	*p*	*Waldχ^2^*	*p*	*Waldχ^2^*	*p*
Intercept	9996.444	<0.001	2766.361	<0.001	2152.317	<0.001	2452.230	<0.001
Group	3.835	0.050	0.152	0.697	0.014	0.907	0.018	0.893
Time	2586.712	<0.001	219.433	<0.001	70.194	<0.001	149.364	<0.001
Group × time	4.373	0.112	8.250	0.016	3.623	0.163	6.158	0.046

**FIGURE 3:**
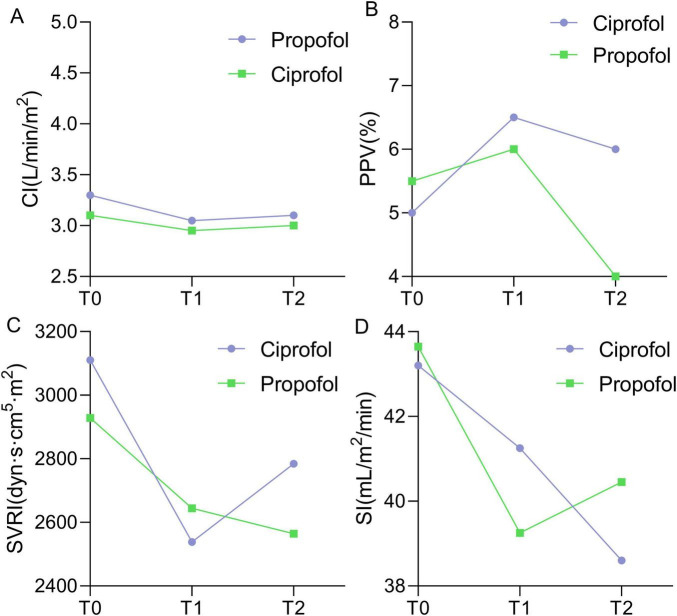
Changes in CI **(A)**, PPV **(B)**, SVRI **(C)**, and SI **(D)** from T0 to T2, with purple for ciprofoll and green for propofol groups.

In both groups, CI, SVRI, and SI decreased at T1 and T2 compared to T0, while PPV initially increased and then decreased (Figure 3). GEE analysis was applied to estimate CI, PPV, SVRI, and SI at different time points, using an exchangeable working correlation matrix. A significant time effect was observed for SVRI and SI in both groups (Table 3), with a significant interaction effect between group and time for SI.

**TABLE 3:** Interaction effects of group and time on CI, PPV, SVRI, and SI in the GEE.

Parameter	CI		PPV		SVRI		SI	
	*Waldχ^2^*	*p*	*Waldχ^2^*	*p*	*Waldχ^2^*	*p*	*Waldχ^2^*	*p*
Intercept	149.325	<0.001	281.342	<0.001	702.486	<0.001	1247.942	<0.001
group	1.134	0.287	0.957	0.328	0.004	0.953	0.007	0.932
time	5.708	0.058	3.179	0.204	19.492	<0.001	7.908	0.019
group × time	1.116	0.572	0.813	0.666	0.082	0.960	11.271	0.004

The time to achieve target anesthesia depth (T1-T0) did not differ significantly between groups (Table 4). The number of patients receiving norepinephrine and the total dosage of norepinephrine were significantly lower in the ciprofol group compared to the propofol group. However, no statistically significant difference was found in the number of patients receiving atropine or in the total dosage of atropine between the two groups.

**TABLE 4:** Comparison of anesthesia depth time and vasopressor dosage.

Indicators	Ciprofol (*n* = 30)	Propofol (*n* = 30)	*Z/χ^2^*	*p*
T1-T0 (s)	66.5 (61.0–122.3)	68.0 (61.0–125.0)	−0.104	0.917
Norepinephrine administration	6	17	8.531	0.003
Atropine administration	1	5	1.667	0.197
Average dosage of norepinephrine (μg)	0 (0–0)	40 (0–80)	−3.142	0.002
Average dosage of atropine (mg)	0 (0–0)	0 (0–0)	−1.707	0.088

There were no statistically significant differences between the two groups in terms of maximum SBP, maximum MAP, maximum DBP, maximum HR, or minimum HR during induction (Table 5). However, the ciprofol group had higher minimum values for SBP, MAP, and DBP compared to the propofol group.

**TABLE 5:** Comparison of maximum and minimum SBP, DBP, MAP, and HR during the induction phase of anesthesia.

Indicators	Ciprofol (*n* = 30)	Propofol (*n* = 30)	*t*	*p*
Maximum SBP (mmHg)	164.90 ± 21.77	170.63 ± 22.72	−0.998	0.322
Minimum SBP (mmHg)	112.93 ± 24.38	95.27 ± 27.58	2.629	0.011
Maximum MAP (mmHg)	112.27 ± 13.52	117.07 ± 18.55	−1.145	0.257
Minimum MAP (mmHg)	77.60 ± 15.75	64.70 ± 13.29	3.430	0.001
Maximum DBP (mmHg)	86.10 ± 12.04	88.30 ± 15.42	−0.616	0.540
Minimum DBP (mmHg)	58.90 ± 14.36	49.63 ± 8.46	3.046	0.004
Maximum HR	85.00 ± 10.19	83.07 ± 12.53	0.656	0.515
Minimum HR	63.73 ± 7.47	61.67 ± 10.95	0.854	0.397

The incidence of hypotension was significantly lower in the ciprofol group (20% vs. 63%, Table 6). Three participants required additional induction drugs: one in the ciprofol group and two in the propofol group.

**TABLE 6:** Adverse events during anesthesia induction.

Indicators	Ciprofol (*n* = 30)	Propofol (*n* = 30)	χ*^2^*	*p*
Movement	1 (3%)	3 (10%)	0.268	0.605
Tearing	0 (0%)	1 (3%)	<0.001	1.000
Hypertension	0 (0%)	1 (3%)	<0.001	1.000
Hypotension	6 (20%)	19 (63%)	11.589	<0.001
Tachycardia	2 (6.7%)	3 (10%)	<0.001	1.000
Coughing	1 (3%)	2 (6.7%)	<0.001	1.000
Additional induction drugs	1 (3%)	2 (6.7%)	<0.001	1.000

## 4 Discussion

This study found that ciprofol is equally effective as propofol for anesthesia induction in elderly patients undergoing lumbar surgery. Both groups achieved comparable anesthesia outcomes; but the ciprofol group exhibited more stable hemodynamics and a significantly lower incidence of hypotension compared to the propofol group. Perioperative hypotension has been robustly associated with myocardial injury, myocardial infarction, and mortality (16, 17). Maintaining hemodynamic stability in elderly patients poses a significant challenge for anesthesiologists, particularly during general anesthesia (18).

Elderly patients experience a decline in physiological reserve and marked alterations in drug metabolism. This can lead to intraoperative hemodynamic instability, such as hypotension, which elevates the risk of adverse outcomes in this vulnerable population (19). The considerable variability in drug metabolism and pharmacodynamics (20), coupled with diminished renal function (21), further complicates the maintenance of stable hemodynamics in elderly patients. When managing anesthesia in this demographic, careful consideration should be given to the selection of anesthetic techniques and agents to optimize efficacy and minimize the risk of complications. Current evidence suggests that ciprofol offers a significant advantage over propofol in maintaining intraoperative hemodynamic stability, positioning it as a promising alternative for general anesthesia in elderly patients.

As an isomer of propofol, ciprofol has been shown to exert a lesser degree of cardiovascular suppression in generally healthy patients (22). Given that hypotension is a common adverse effect during propofol induction, ciprofol is gaining attention as a promising induction agent, particularly in elderly patients. In robust patients undergoing elective general anesthesia for gynecological surgery, a ciprofol dose of 0.4 mg/kg demonstrated the same success rate for anesthesia induction as 2 mg/kg of propofol (8). Meta-analyses have shown that ciprofol has similar efficacy to propofol for anesthesia induction while presenting a lower risk of hypotension and injection pain (23). Furthermore, the dose-response relationship for anesthetic agents exhibits unique characteristics in elderly patients. Owing to decreased drug distribution volume and clearance (21), the same dose of an anesthetic may have enhanced effects in this population. Duan et al. (24) found that a dose of 0.3 mg/kg of ciprofol provided optimal safety and sedation depth during anesthesia induction in elderly patients, effectively maintaining hemodynamic stability with minimal adverse events. Conversely, a dose of 0.2 mg/kg of ciprofol resulted in shallower sedation with a higher need for rescue sedation, while a dose of 0.4 mg/kg raised the risk of hypotension and bradycardia, albeit with successful induction across all doses tested.

Based on the studies by Luo et al. (25) and Zeng et al. (26), and in accordance with the recommended dosages in the drug labeling, we adopted a baseline ciprofol dose of 0.4 mg/kg and a propofol dose of 2 mg/kg in the current study, with adjustments tailored to individual patient health status. Our results demonstrated successful general anesthesia induction in all patients. While blood pressure trends were comparable between groups, the incidence of hypotension was significantly lower in the ciprofol group than in the propofol group, as was the requirement for norepinephrine. A significant group-time interaction effect was observed for SBP, MAP, and SI, indicating more pronounced hemodynamic fluctuations and larger deviations in the propofol group relative to the ciprofol group. These findings underscore ciprofol’s superiority in maintaining hemodynamic stability during anesthesia induction in elderly patients. In summary, ciprofol’s unique pharmacokinetic characteristics appear to confer distinct advantages in anesthesia management for the elderly. A phase 2 clinical trial reported that ciprofol has a shorter half-life and lower volume of distribution compared to propofol (23), which may explain its enhanced hemodynamic stability.

A recent pharmacokinetic study (27) in elderly patients found that ciprofol had a comparable elimination half-life (3.47 vs. 2.85 h) and volume of distribution (3.96 vs. 3.18 L/kg) to propofol, but significantly slower clearance (0.83 vs. 1.52 L/h/kg). This is consistent with the meta-analysis, which reported no significant difference in hypotension risk between ciprofol and propofol (28). However, ciprofol significantly reduced respiratory depression and hypoxemia. Jin et al. (29) found that in outpatient hysteroscopy, etomidate (0.4 mg/kg) more effectively suppressed procedural responses while exhibiting significantly lower rates of respiratory depression, hypoxemia, and injection pain than propofol (2 mg/kg). Hung et al. (23) further confirmed that while both agents provide comparable sedation and anesthetic induction, etomidate presents a reduced risk of hypotension and injection pain. Additionally, Hudaib et al. (30) highlighted etomidate’s superior pain management during induction, contributing to enhanced patient comfort. These research results consistently support the significant advantages of ciprofol in reducing respiratory depression, hypotension, and injection pain, indicating that its application in clinical anesthesia has higher safety and patient comfort.

Several factors may influence the outcomes observed in this study. Elderly patients exhibit age-related physiological changes, including reduced cardiovascular compensatory mechanisms and altered pharmacokinetics, which increase susceptibility to hemodynamic instability during anesthesia induction (4–6, 12). Additionally, lumbar surgery requires prone positioning, which can exacerbate venous pooling and reduce cardiac preload, further contributing to hypotension (11). Comorbidities (e.g., hypertension, coronary artery disease) and preoperative medications may also modulate drug responses. For instance, patients with ASA III status in this study had higher baseline cardiovascular risk, which may have amplified the differences in hypotension rates between ciprofol and propofol groups. Future studies should stratify patients by comorbidity severity and medication use to explore these interactions in depth.

This study has several limitations. First, the exclusive use of a 0.4 mg/kg ciprofol dose precludes evaluation of potential dose-response relationships. Second, the relatively modest sample size and short observation period may restrict the ability to detect rare adverse events or long-term outcomes. Notably, the study excluded patients with a BMI ≥28 kg/m^2^, a threshold based on China-specific BMI classifications where this value is considered obese. Drug distribution and metabolism in obese patients often differ from those in patients with normal BMI, and fixed dosing regimens may not fully reflect the efficacy and safety profiles of drugs across varying BMI populations. Obese patients are potentially more susceptible to postoperative complications or residual drug effects, which may not be adequately captured within a limited observation window. Given these factors, the findings of this study may not be directly applicable to patients with BMI ≥28 kg/m^2^. Future studies should consider including a wider BMI range to assess the potential impact of obesity on anesthetic effects and related complications, which would provide more comprehensive clinical data. Furthermore, the injection pain associated with propofol, as reported in previous studies, was not observed in this study, possibly due to the sample size limitations. The relatively small sample size (*n* = 60) and short observation period may restrict the ability to detect rare adverse events or long-term outcomes. Larger multicenter trials with extended follow-up are warranted to validate these findings and assess long-term safety profiles, especially in populations with diverse comorbidities or body mass indices.

In conclusion, For elderly patients undergoing lumbar surgery, a dose of 0.4 mg/kg of ciprofol is recommended for anesthesia induction, as it provides comparable efficacy to propofol while significantly reducing the incidence of hypotension. Ciprofol may be particularly beneficial for elderly patients with a history of cardiovascular disease or those at higher risk of hypotension. However, individual patient factors should be considered, and the decision to use ciprofol should be made on a case-by-case basis.

## 5 Conclusion

In conclusion, ciprofol demonstrated comparable anesthetic efficacy to propofol for anesthesia induction in elderly patients undergoing lumbar surgery, with more favorable hemodynamic effects and a lower incidence of hypotension. Specifically, ciprofol at a dose of 0.4 mg/kg showed significantly lower hypotension incidence (20% vs. 63%) and reduced norepinephrine use compared to propofol at 2 mg/kg during induction, addressing a critical concern in geriatric anesthesia. Both agents achieved comparable anesthesia depth (time to target BIS ≤ 60) and intubation conditions, validating ciprofol’s clinical utility in this population. The group-time interaction effect on SBP and mean MAP suggests that ciprofol mitigates hemodynamic fluctuations more effectively than propofol. These findings highlight ciprofol’s potential as a safer and more effective alternative for anesthesia induction in elderly patients undergoing lumbar surgery.

## Data Availability

The data analyzed in this study is subject to the following licenses/restrictions: The datasets presented in this article are not readily available because of ethical concerns, the data has potentially identifiable information. Requests to access these datasets should be directed to YC, 402009694@qq.com.
